# Ultrasensitive Microfiber Biosensor with Synergistic Sensitization of Gold Nanoparticles and Two-Dimensional Material Black Phosphorus for Detection of BRCA1 Gene Synthetic Sequence

**DOI:** 10.3390/bios16030165

**Published:** 2026-03-16

**Authors:** Lina Wang, Duo Yi, Youfu Geng, Xuejin Li, Chong Li, Junyu Niu

**Affiliations:** 1 College of Electronic Engineering, Huainan Normal University, Huainan 232038, China; wanglina2017@email.szu.edu.cn; 2 Shenzhen Key Laboratory of Sensor Technology, Shenzhen University, Shenzhen 518060, China; yiduo@szu.edu.cn (D.Y.); gengyf@szu.edu.cn (Y.G.); lixuejin@szu.edu.cn (X.L.); 3 School of Science and Engineering, Chinese University of Hong Kong, Shenzhen 518172, China; 4 Shenzhen Key Laboratory of Advanced Thin Films and Applications, College of Physics and Optoelectronic Engineering, Shenzhen University, Shenzhen 518060, China; 5 Xi’an Structure-Function Materials International Science and Technology Cooperation Base, School of Materials and Chemical Engineering, Xi’an Technological University, Xi’an 710021, China

**Keywords:** breast cancer genes, gold nanoparticles, black phosphorus nano-interface, signal amplification, microfiber biosensor

## Abstract

Sensitive quantitative detection of breast cancer gene synthetic sequences is crucial for related biosensing research. To address the limitations of traditional sensors for detecting ultra-low concentrations, this study developed a novel fiber-optic biosensor by combining nanomaterial sensitization with nanoparticle signal amplification strategies. A fiber optic sensor based on single-mode fiber-thin-core fiber-multimode fiber-single-mode fiber structure was fabricated and functionalized with black phosphorus (BP) nano-interface. The Au@cDNA complex was prepared by covalently immobilizing sulfhydryl-modified complementary DNA (cDNA) on the surface of gold nanoparticles (AuNPs). The complex specifically hybridized with the probe DNA (pDNA) immobilized on the surface of the sensor. The experimental results show that this sensor has a sensitivity of 0.793 nm/lgM and a detection limit of 20.27 fM in the concentration range of 100 fM to 100 nM. Specifically, the BP-functionalized sensor exhibits superior dynamic range, higher sensitivity, and lower detection limits for detecting Au@cDNA. The synergistic effect of interfacial sensitization by BP and signal amplification by AuNPs significantly enhances detection performance, providing a promising platform for ultra-sensitive biosensing applications.

## 1. Introduction

Breast cancer is one of the major malignant tumors threatening women’s health, ranking among the top in global incidence and mortality rates among all cancers. In recent years, the age of diagnosis has shown a trend toward younger patients [1]. Early-stage breast cancer typically presents no obvious clinical symptoms, leading to most patients being diagnosed only after the disease has progressed to an advanced stage. Early detection of breast cancer is crucial for improving cure rates and reducing mortality. Although X-ray screening has been shown to be effective in reducing breast cancer mortality in women over 50 years of age, its applicability to young women is limited [2,3]. Therefore, the development of a detection method with high sensitivity, simple operation, and low cost has become a research hotspot in nucleic acid analysis. In recent years, gene detection technologies based on biosensors have provided a new approach for early diagnosis and risk assessment of cancer [4]. Furthermore, genetic testing has shown broad application prospects in promoting precision medicine, drug development, genetic counseling, and forensic identification.

Genetic risk testing for breast cancer primarily focuses on analyzing genes such as BRCA1, BRCA2, and HER2. BRCA1 is a key breast cancer susceptibility gene, and its normal function is related to tumor suppression [5,6,7]. Changes in this gene significantly increase the risk of developing familial breast cancer. Studies indicate that approximately 40–50% of hereditary breast cancer cases are associated with the BRCA1 gene sequence. Some people may also have an increased risk of ovarian cancer [8]. Therefore, BRCA1 gene testing can be used to assess an individual’s genetic predisposition to breast and ovarian cancer [9]. Currently, the measurement techniques for the BRCA1 gene include single-strand conformation polymorphism analysis, DNA sequencing, polymerase chain reaction, and real-time fluorescence quantitative analysis. These methods are relatively mature and have been well supported by the extensive literature [10,11]. However, they typically require substantial sample volumes, involve lengthy testing cycles, and carry high costs. Therefore, developing a DNA sequence detection method with high sensitivity, high specificity, and low cost is of great significance for biomedical research and disease prevention and control.

Electrochemical DNA biosensors have shown significant application value in the early detection of breast cancer due to their high sensitivity, rapid response, and excellent selectivity [12,13,14,15]. In 2014, Rasheed et al. proposed a graphene-based electrochemical DNA sensor. This sensor adopts a sandwich-type detection mode and utilizes gold nanoparticles to achieve signal amplification. The detection limit for the BRCA1 gene reached the femtomolar (fM) level [16]. Although electrochemical methods have advantages in terms of sensitivity, their electrodes are usually large in size, which is not conducive to the miniaturization and integration of devices. Considering requirements such as structural flexibility, detection limits, response speed, reliability, and biocompatibility, fiber-optic sensors demonstrate promising development prospects.

Various fiber-sensing mechanisms, such as long-period fiber gratings (LPGs), tilted Bragg fiber gratings (TFBGs), and fiber interferometric measurement techniques have been employed for HER2 detection research [17,18,19]. In 2020, Sun et al. proposed a compact biosensor based on a tapered fiber interferometer for detecting the HER2 biomarkers [20]. This sensor reduced the fiber diameter to 8 μm, achieving a sensitivity of 1867 nm/RIU, and enabled detection of 2 ng/mL HER2 in serum through surface-immobilized antibodies. While the tapered structure enhances the optical field and improves sensitivity, its low mechanical strength limits its applicability in certain environments.

At present, most reports on the application of optical fiber sensors in breast cancer are limited to HER2 detection, and there are few studies involving BRCA1 sequence detection. Therefore, this study designed an optical fiber biosensor based on the Mach-Zehnder interference (MZI) principle. By functionalizing the surface of the fiber, BRCA1 synthetic sequence probes were immobilized in the sensing area. This probe can form a double-stranded structure with the target complementary DNA through specific hybridization, thereby enabling the detection and analysis of the synthetic sequences. To improve the detection sensitivity of low-concentration DNA sequences, two strategies were adopted in this study: one is to use complementary DNA modified with gold nanoparticles (AuNPs) for signal amplification; the other is to introduce a two-dimensional material, black phosphorus (BP), to enhance the response performance of the sensor. The sensor achieved a detection limit at the fM range, providing a highly sensitive novel approach for specific gene sequence analysis. At this stage, this study employs synthetic sequences to evaluate sensor performance as a preliminary exploration. Subsequent studies will be conducted to further optimize the sensor and expand its application scope.

## 2. Operating Principle and Sensor Fabrication

**Figure 1:**
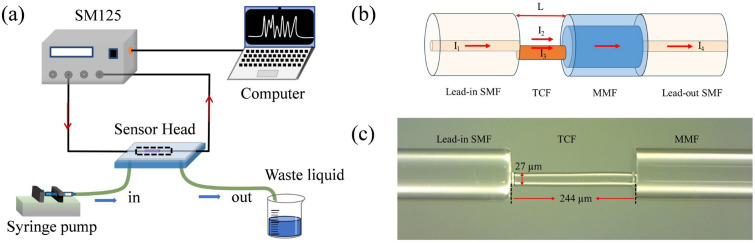
(**a**) Diagram of the sensing system for DNA detection. (**b**) Schematic diagram of an optical fiber biosensor based on the MZI structure. (**c**) Optical microscope of the sensor.

The schematic diagram of the experimental system for detecting DNA is shown in Figure 1a. It mainly consists of a fiber-grating sensing demodulator (SM125, wavelength-scanning range 1510–1590 nm, Micron Optics Co., Ltd., Atlanta, GA, USA), a computer, a syringe pump, a beaker, and a sensing probe. The fiber grating sensing demodulator integrates a scanning laser source and a data acquisition module, and can be directly connected to both ends of the sensing probe via the optical fiber port. During the detection process, the solution containing different concentrations of target DNA was continuously injected into the microfluidic channel by a syringe pump at a constant flow rate. After each test, the waste solution is extracted from the outlet and collected in a beaker. All the tests at different concentrations were independently repeated at least three times under the same conditions to evaluate the repeatability and stability of the measurement results. Finally, the experimental data were transmitted to the computer for subsequent analysis and processing. After each detection, the sensor was immersed in the piranha solution to remove the surface-bound analyte, and then the same sensor was used for subsequent detection cycles.

Figure 1b shows a schematic diagram of the MZI sensing structure based on SMF-TCF-MMF-SMF, abbreviated as STMS. The fabrication of the sensing probe mainly includes two steps: drawing the TCF and structure splicing. Among them, the TCF cannot be purchased directly from the company but is drawn from a coreless optical fiber with a diameter of 125 µm. The detailed drawing process has been provided in our previous work [21]. The splicing process of the sensor is also relatively simple, requiring a fiber fusion splicer (Fujikura FSM-100P+, Fujikura Ltd., Tokyo, Japan) and a fiber cleaver, as follows: Firstly, a section of 125 µm diameter MMF and a section of 125 µm diameter Lead-out SMF are fusion-spliced directly. The arc power and continuous discharge time of the fusion splicer were set to 290 bits and 2000 ms, respectively. Secondly, one end of the prepared TCF was spliced with MMF-SMF to obtain the TCF-MMF-SMF structure. Here, in order to obtain interference fringes with high extinction ratios, the fusion parameters of the fiber fusion splicer are optimized. The Y-direction needs to be set to a lateral offset of 13.5 µm; the X-direction is lateral alignment; and the arc power and duration are 480 bits and 300 ms, respectively. Finally, the STMS structure was constructed by splicing the TCF with the Lead-out SMF after cutting the TCF to retain a length of about 244 µm.

The operating principle of the sensor is that when the scanning light (I_1_) output from the fiber grating demodulator reaches the TCF through the Lead-in SMF, the incident light beam is divided into two parts: I_2_ and I_3_. I_2_ propagates in the air, and I_3_ propagates along TCF. I_2_ and I_3_ are recoupled into the MMF, and I_4_ is output through the Lead-out SMF. Due to the different transmission paths of these two beams, an MZI will be generated. In addition, MMF is employed to enhance the coupling efficiency between TCF and SMF, which, in turn, optimizes the insertion loss and contrast of the interference spectrum. Figure 1c shows the optical microscope image of the sensor structure. It can be seen that the prepared TCF has a diameter of only 27 µm and a length of 244 µm. Compared with common SMF/MMF, the advantage of TCF is that its surface can directly excite the evanescent wave, which significantly improves the interaction efficiency with the surrounding environment.

**Figure 2:**
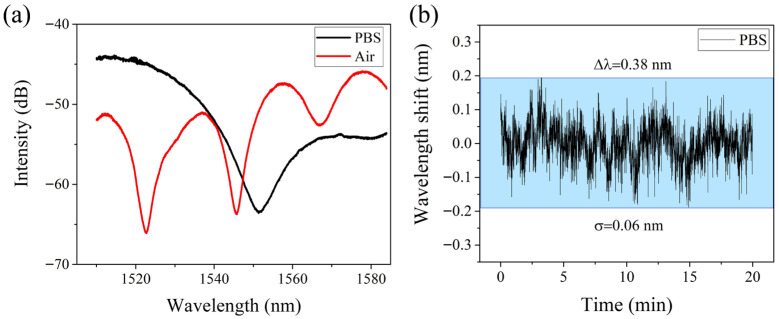
(**a**) Transmission spectra of the optical fiber sensor in air and PBS. (**b**) Wavelength fluctuation test of the sensor.

The working principle of the sensor is Mach-Zehnder interference (MZI) based on optical fiber sensing. Figure 2a shows the transmission spectra of the optical fiber sensor based on the STMS structure in air and PBS, respectively. In the experiment, the wavelength change of the selected trough is recorded in real time through the peak-tracking function of SM125. The wavelength-stability test results for this sensor are shown in Figure 2b. The resonance wavelength of the sensor was continuously monitored for 20 min in PBS buffer solution. The total wavelength shift was 0.38 nm, and the standard deviation was 0.06 nm, indicating that the sensor had good wavelength stability.

## 3. Experimental Reagents and Preparation of Au@DNA

### 3.1. Experimental Reagents

The following reagents are prepared in the experiment: phosphate buffer solution (PBS, pH = 7.2–7.4), 98% concentrated sulfuric acid, 30% hydrogen peroxide solution, Poly-L-Lysine (PLL, molecular weight of 150,000~300,000 g/mol), sodium chloride (NaCl), 98% 3-aminopropyltriethoxysilane (APTES), absolute ethanol, deionized water, two-dimensional nanomaterial black phosphorus (BP), and gold nanoparticles (AuNPs, 10 nm in diameter). BP and AuNPs are purchased from Nanjing XFNANO Materials Tech Co., Ltd. (Nanjing, China). The BRCA1 synthetic sequences are purchased from Shanghai Sangon Biotech Co., Ltd. (Shanghai, China). Table 1 lists the 40-base oligonucleotide probe DNA (pDNA), sulfhydryl-modified target complementary DNA (cDNA), and sulfhydryl-modified non-complementary target DNA (N-cDNA). Fetal bovine serum (FBS) is purchased from Shanghai Macklin Biochemical Technology Co., Ltd. (Shanghai, China). Bovine serum albumin (BSA, concentration: 0.1 mg/mL) is purchased from Sigma-Aldrich (St. Louis, MO, USA), which conforms to the standards of molecular biology and immunology experiments.

BP is sensitive to oxygen and water under environmental conditions, and its chemical degradation is indeed one of the main challenges in the application of this material. As a sensitizing layer, BP is directly exposed toa liquid environment, and its stability needs to be considered. Studies have shown that the degradation rate of BP is closely related to environmental pH and is relatively stable in a neutral pH environment [22]. In our sensing experiment, BP was in contact with PBS buffer/test solution. The pH value of the experimental environment was controlled between 7.2 and 7.4, which slowed down the hydrolysis and oxidation rate to a certain extent. BP must be diluted with isopropanol prior to use, stored at 4 °C protected from light, and it is recommended to be used within 15 days.

### 3.2. Preparation of Au@DNA

Compared with other nanomaterials, AuNPs possess distinctive advantages, including facile synthesis, excellent biocompatibility, and low production costs, which have established their prominent role in biomedical research and applications. It is worth noting that AuNPs provide significant signal enhancement in specific DNA-sequence analysis applications, allowing ultra-sensitive detection of target molecules at trace levels. Various methods have been reported for the preparation of Au@cDNA complexes, mainly salt aging [23], freezing [24], low pH [25], and butanol dehydration [26]. Among them, the salt aging method is the preferred approach for preparing Au@cDNA.

**Figure 3:**
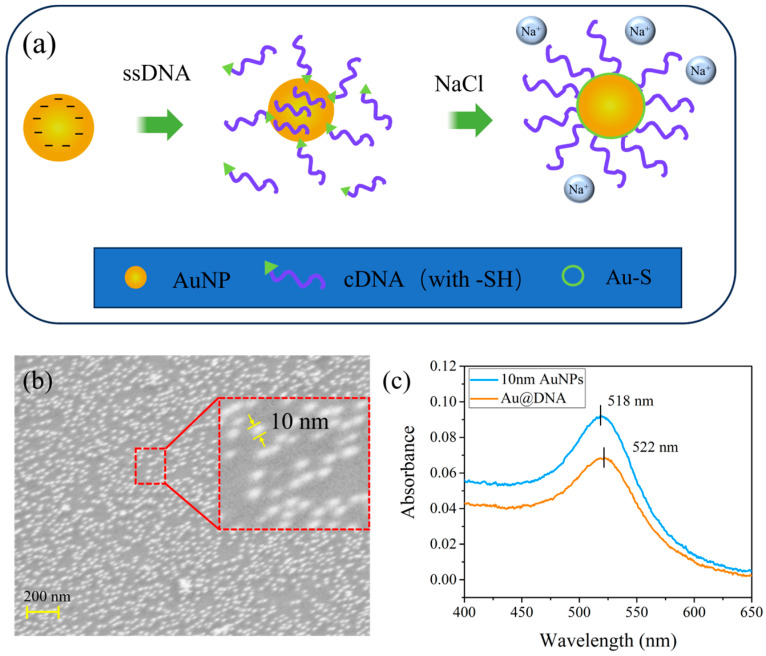
(**a**) Schematic diagram of cDNA binding to AuNPs. (**b**) SEM image of AuNPs. (**c**) UV absorption spectra of AuNPs and Au@DNA.

Since DNA itself is negatively charged, a small amount of DNA modification on the surface of AuNPs increases the negative charge density on its surface. This increased charge density generates electrostatic repulsion that inhibits further binding of free sulfhydryl-modified DNA strands to the surface of AuNPs, and thus the DNA is unable to densely arrange on the surface. To address the low DNA binding efficiency on AuNPs, salts (e.g., NaCl) must be added to increase the ionic strength of the solution. This process is also referred to as “aging.” In addition, the DNA monolayer is more stable due to the strong interaction of the Au-S covalent bond. Figure 3a shows the schematic diagram of the salt aging method for high-density accumulation of DNA on the surface of AuNPs in the following steps:

(1) The cDNA with modified sulfhydryl groups is mixed with AuNPs (aqueous solution) and left at 4 °C for 16 h under light protection.
(2) 20× PBS buffer is added every 15 min dropwise to change the concentration of NaCl in the mixed solution to 0.05 M and left to stand for 6 h. The mixed solution was then incubated at 4 °C (protected from light).
(3) Repeat the above steps until the concentration of NaCl in the mixed solution reaches 0.1 M, then leave for 6 h.
(4) The mixed solution is finally diluted to 1× PBS buffer solution and stored in a refrigerator at 4 °C. The Au@cDNA solution prepared in this way can be stored for several months.

The Scanning electron microscopy (SEM) image of AuNPs is shown in Figure 3b. It can be seen that the AuNPs are spherical and well dispersed with an average particle size of about 10 nm. The UV-visible spectra of AuNPs before and after DNA modification are shown in Figure 3c. The experimental results show that the absorption peak of AuNPs is close to 518 nm, as shown in the blue curve. The absorption peak of Au@DNA after DNA modification is red-shifted to 522 nm, as shown in the orange curve. This wavelength shift of about 4 nm is mainly due to the DNA biomolecules altering the volume of AuNPs and the RI of the medium surrounding the AuNPs, which also fully confirms the successful functionalization of DNA modification on the surface of AuNPs.

## 4. Detection of Au@cDNA

**Figure 4:**
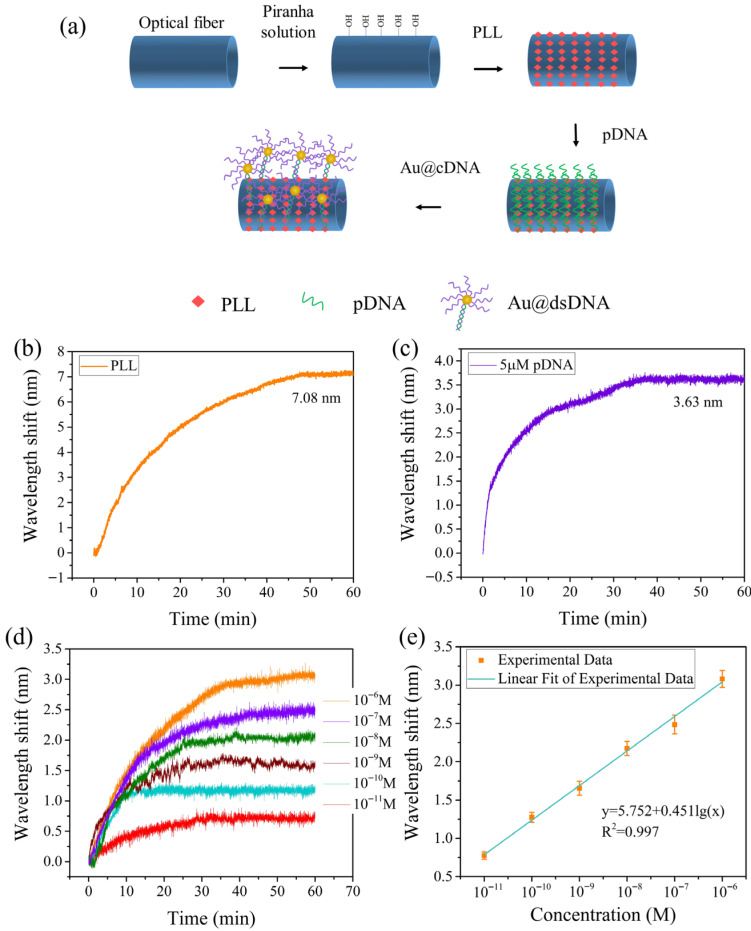
(**a**) The detection schematic diagram of Au@cDNA. (**b**) Spectral wavelength shift of the PLL biofilm layer modified on the surface of the micro-structured fiber. (**c**) Wavelength shift of 5 µM pDNA binding. (**d**) Wavelength response of the sensor to different concentrations of Au@cDNA. (**e**) The relationship between wavelength shift and different concentrations of Au@cDNA.

The surface functionalization step and the Au@cDNA binding process are shown in Figure 4a. The sensitivity of the sensor is directly determined by the effective RI difference between the sensing arm (analyte solution) and the reference arm (waveguide mode in TCF). It should be noted that the light from the other parts of the sensing structure, such as Lead-in SMF, MMF, and Lead-out SMF, is tightly confined to the fiber core, so the combination of any biomolecules in these parts has little effect on the interference spectral shift. The wavelength shift we measured mainly comes from the binding events on the TCF surface.

(1) Sensor surface pretreatment: firstly, the surface of the fiber optic sensor is cleaned using deionized water and ethanol.
(2) Surface hydroxyl activation: The clean fiber was immersed in Piranha solution (concentrated sulfuric acid to 30% hydrogen peroxide 3:1 by volume) for 30 min to activate the surface hydroxyl groups (-OH). After treatment, the fiber is rinsed well with deionized water and dried at room temperature.
(3) Poly-L-Lysine (PLL) modification layer deposition: Following this, the PLL solution was injected into the flow cell and incubated for 1 h at room temperature to form a biocompatible modification layer on the surface of the optical fiber by electrostatic adsorption.
(4) Binding of probe DNA (pDNA): Subsequently, 5 µM solution of pDNA was injected into the flow cell and fixed for 1 h at room temperature. After immobilization, the flow cell was rinsed with phosphate buffer solution (PBS, pH 7.4) for 5 min to remove unbound pDNA molecules.
(5) Sensing performance characterization: Finally, the performance parameters of the functionalized fiber optic sensors were systematically evaluated by the Au@cDNA hybridization experimental system, including sensitivity, detection range and limit of detection (LOD).

Figure 4b shows the wavelength shift caused by the binding of PLL to the fiber surface. The wavelength of the transmission spectrum changes with time and tends to be stable after 50 min. The total wavelength shift is 7.08 nm within 60 min. Figure 4c shows the real-time wavelength response curve of the pDNA with a concentration of 5 µM in the process of binding to the PLL layer by electrostatic attraction. The continuous binding of pDNA on the fiber surface increases the local RI, causing the wavelength to shift. The response curve indicates a rapid and significant wavelength shift within the first 20 min, followed by a gradual decrease in the shift rate until stabilization. A total wavelength shift of about 3.63 nm was observed over the 60 min monitoring period. The stabilization of the spectral response indicates the completion of sensor surface functionalization.

To evaluate the signal enhancement effect of AuNPs, the functionalized sensor was placed in Au@cDNA solutions with concentrations ranging from 10 pM to 1 µM. The wavelength shifts of the interference spectra at different concentrations are shown in Figure 4d. As the Au@cDNA concentration gradually increased from 10 pM, the wavelength shift increased significantly. Each concentration point was tested three times. The relationship between the wavelength shift and the concentration is shown in Figure 4e. In the range of 10 pM to 1 µM, the detection sensitivity of the sensor to Au@cDNA was 0.451 nm/lgM (i.e., nm per log_10_(M)), and the linear correlation coefficient was 0.997. According to the triple standard deviation and the fitting equations in Figure 4e, the detection limit of the sensor was calculated to be 0.448 pM. The detection limit was reduced by about 35.2 times compared to the detection results previously obtained without using AuNPs [21]. The above results show that the AuNPs signal amplification strategy effectively improves the sensitivity of the sensor and provides a potential technical approach for the detection of low-concentration biomarkers.

## 5. Performance of BP Functionalized Sensor

**Figure 5:**
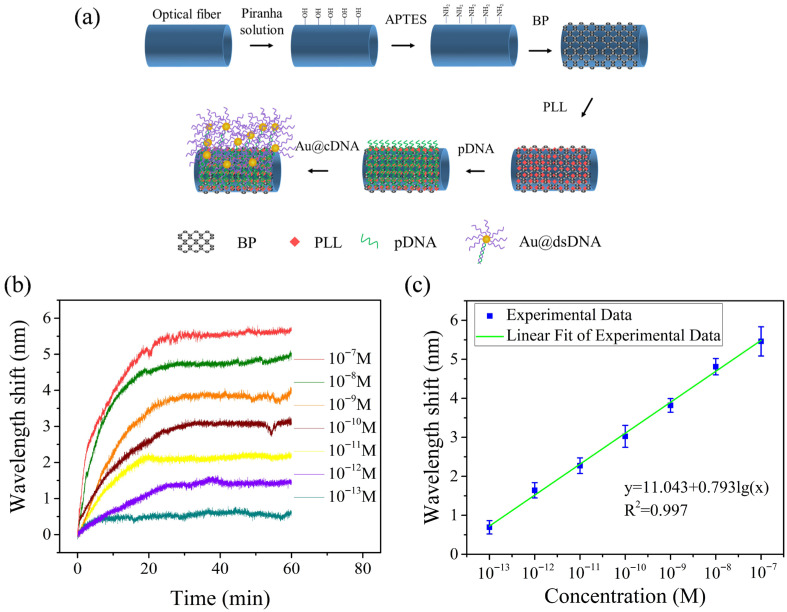
(**a**) Schematic diagram of functionalization of the optical fiber surface with BP and biological molecule binding of Au@cDNA. (**b**) With the increase of DNA concentration from 100 fM to 100 nM, the wavelength of the interference spectrum changes. (**c**) Relationship between wavelength shift and Au@cDNA concentration.

In order to further optimize sensing performance, we used BP nanosheets to sensitize the optical fiber sensing interface. The related modification steps and Au@cDNA detection process are shown in Figure 5a. It is worth noting that the following two steps need to be added compared to the comparison test of the bare sensor without BP.

(1) The optical fiber treated by Piranha solution was immersed in APTES solution for 1 h. Subsequently, it was fully cleaned with deionized water to remove non-covalently adsorbed silane molecules, thereby introducing an amino functional group (-NH_2_) on the surface of fiber.
(2) The above functionalized fiber was fixed on a clean glass substrate, and 10 µL BP dispersion (solvent is isopropanol) was added dropwise in the sensing area three times. Dry at room temperature for 10 min after each drop. This layer-by-layer deposition method helps to enhance the adhesion stability of BP nanosheets on the fiber surface.

In addition, we tested the BP-sensitized fiber biosensor at different concentrations of the Au@cDNA solution to evaluate the performance of the sensor. The specific binding between the target molecule and the probe results in a corresponding shift in the transmission spectrum of the sensor, as shown in Figure 5b. It can be seen that the wavelength offset gradually increases with the increase of Au@cDNA concentration. In order to ensure the reliability of the data, at least three independent repeated experiments were carried out at each concentration point, and the experimental conditions were strictly consistent with the operational process.

As shown in Figure 5c, the wavelength shift of the interference spectrum has a good linear relationship with the logarithm of Au@cDNA concentration in the range of 100 fM to 100 nM. By calculating the average value and error bar of the spectral wavelength shift data at each concentration gradient, linear fitting can be obtained. The spectral response sensitivity of the sensor modified with BP to Au@cDNA is 0.793 nm/lgM, the linear correlation coefficient is 0.997, and the LOD reaches 20.27 fM (the calculation method is the same as above). The experimental results show that the optical fiber biosensor has good sensitivity.

As shown in Table 2, the STMS fiber-optic sensor proposed in this study has a detection limit (20.27 fM) at the same order of magnitude as the electrochemical method based on AuNPs signal amplification (1 fM) [27] and is significantly superior to other electrochemical sensing platforms [28,29,30]. More importantly, the sensor adopts the principle of optical interference detection and has the unique advantages of a simple structure, miniaturization, anti-electromagnetic interference, and reusability. Although the current detection object is a synthetic sequence, the platform provides a competitive technical solution for high-sensitivity detection of BRCA1-related genes in biomedical research.

## 6. Specific Detection

**Figure 6:**
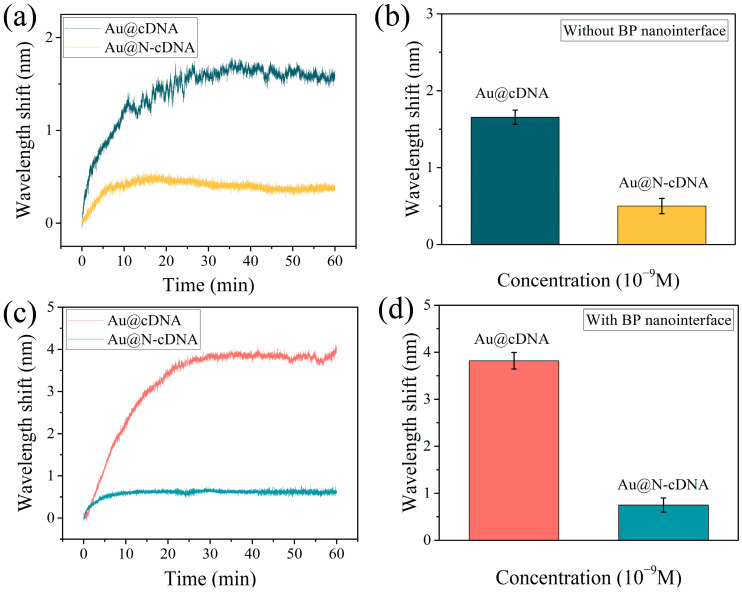
(**a**) The real-time wavelength response of the bare optical fiber sensor to Au@cDNA and Au@N-cDNA. (**b**) Comparison results of the wavelength shift. (**c**) The real-time wavelength response of the BP-modified sensor. (**d**) The corresponding wavelength shift of the BP-modified sensor.

The selectivity of the optical fiber biosensor was confirmed by comparing responses to Au@cDNA and Au@N-cDNA, as shown in Figure 6. The solution concentrations of Au@cDNA and Au@N-cDNA used in the experiment were 1 nM. First, the real-time response curve of the bare fiber sensor without BP deposition is shown in Figure 6a. The wavelength shift caused by Au@cDNA was significantly greater than that caused by Au@N-cDNA. The wavelength shift caused by the interaction of the sensor with the two solutions is shown in Figure 6b. The wavelength shift of Au@cDNA is 1.65 nm, which is 3.3 times that of Au@N-cDNA (0.50 nm). To further enhance detection performance, we modified the surface of the optical fiber with BP and evaluated its specificity.

The real-time response curves and corresponding wavelength shifts of the BP-functionalized sensors are shown in Figure 6c,d. The average wavelength shift for Au@cDNA is 3.82 nm, which is 5.09 times that of Au@N-cDNA (0.75 nm). Experimental results confirm that this fiber biosensor exhibits highly specific recognition of cDNA sequences. By introducing BP nanosheets, the selectivity was further enhanced. This was mainly due to the abundant surface functional groups of BP, which facilitated the effective enrichment and binding of cDNA molecules.

**Figure 7:**
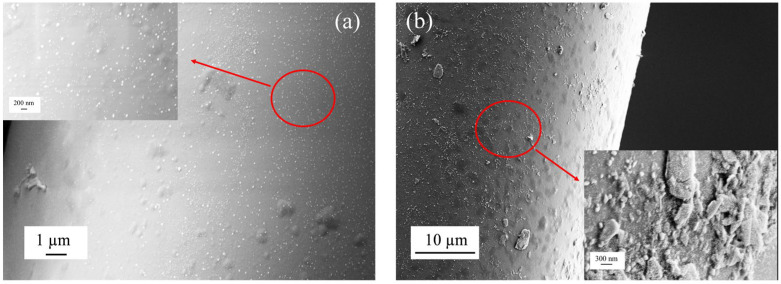
(**a**) SEM images of PLL/pDNA/Au@cDNA. (**b**) SEM images of APTES/BP/PLL/pDNA/Au@cDNA, respectively. Insets show the magnified image of AuNPs.

Figure 7 shows the surface morphology of the bare fiber and the BP functionalized fiber sensor combined with Au@cDNA. As shown in Figure 7a, the surface of the fiber is relatively smooth. A large number of AuNPs are evenly distributed on the surface of the fiber, indicating that the pDNA successfully fixed and captured the Au@cDNA. As shown in Figure 7b, the surface roughness of the fiber modified by BP nanomaterials is significantly increased. More dense gold nanoparticles were clearly observed on the surface, indicating that the BP interface further enhanced the immobilization efficiency of Au@cDNA. The results show that the biomolecules achieve DNA-specific hybridization on the surface of the sensor, which provides a reliable basis for the wavelength change of the interference spectrum.

**Figure 8:**
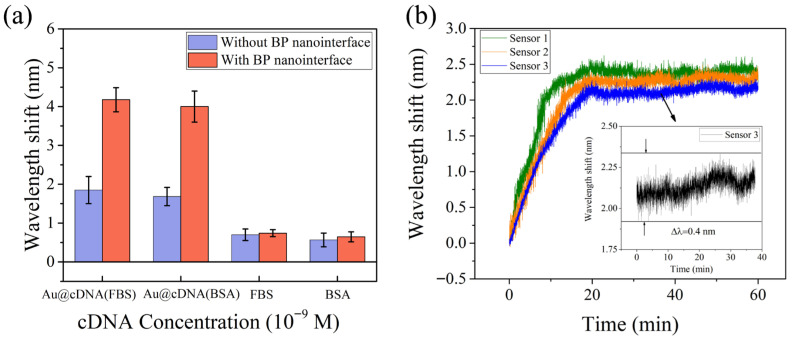
(**a**) Comparison of wavelength shifts of the sensor in the presence of interfering molecules. (**b**) The real-time wavelength shifts response curves of three independent sensors.

Figure 8a shows the specific response of the STMS-based fiber optic biosensor to Au@cDNA in a complex biological sample matrix. In the experiment, fetal bovine serum (FBS, diluted 100 times) and bovine serum albumin (BSA, 0.1 mg/mL) were used as typical biological matrix interfering substances, and the effects of FBS and BSA on the detection accuracy of the sensor were evaluated. The effect of interfering biomolecules on the detection accuracy of the sensor was evaluated by adding 1 nM Au@cDNA to FBS and BSA. The wavelength shifts of bare fiber in FBS and BSA solutions containing Au@cDNA were 1.85 nm and 1.68 nm, respectively. In the FBS/BSA solution, the wavelength shifts were only 0.70 nm and 0.56 nm. In addition, the wavelength shifts of BP functionalized sensors in the above four solutions were 4.17 nm, 4.05 nm, 0.74 nm and 0.64 nm, respectively. The results show that the sensor can still maintain a significant response signal to Au@cDNA in the presence of interference molecules, and the signal is significantly higher than the background response caused by the interference alone. This fully proves that the sensor still has good selectivity and anti-interference ability in the FBS/BSA solution and provides an experimental basis for its application in a complex biological sample analysis.

By employing a standardized fabrication process, we prepared three independent sensors under identical conditions and evaluated their performance in detecting Au@cDNA at concentrations of 1 × 10^−11^ M. Figure 8b shows the real-time wavelength-shift response curves of the three independent sensors (sensor 1, 2, 3). It can be clearly seen from the figure that the three sensors show a highly consistent dynamic response trend. The wavelength shift increased rapidly in the first 20 min and gradually stabilized after about 30 min. The total wavelength shifts for the three tests were 2.43 nm, 2.31 nm, and 2.14 nm, respectively, and the maximum difference of wavelength shift was 0.29 nm. The relative standard deviation (RSD) of the steady-state response was calculated to be 6.35%, confirming the repeatability between the independent probes.

As shown in the inset of Figure 8b, during the monitoring time of 40 min, the wavelength shift of sensor 3 after reaching a stable state is 0.4 nm. The wavelength change is small, indicating that the optical fiber sensing platform runs stably during the experimental period and can meet the detection requirements. In future work, the long-term stability of the sensor will be systematically studied to achieve a more comprehensive performance evaluation.

## 7. Conclusions

This study successfully developed a microfiber biosensor based on signal amplification by AuNPs, enabling highly sensitive detection of the BRCA1 gene synthetic sequences. By combining AuNPs with cDNA to form Au@cDNA complexes and utilizing the two-dimensional material BP as a nano-interface sensitization layer, the detection performance of the sensor was significantly enhanced. Experimental results demonstrate that the bare fiber optic sensor achieves a detection sensitivity of 0.451 nm/lgM and LOD of 0.448 pM under the signal amplification effect of AuNPs. After adopting the BP nanointerface, the sensor achieved a sensitivity of 0.793 nm/lgM and LOD as low as 20.27 fM. The sensor exhibited high selectivity and reliability in specificity experiments, showing significant response differences between complementary and non-complementary DNA, while maintaining stable detection performance even in complex biological matrices such as fetal bovine serum. In summary, this study confirms the signal amplification effect of AuNPs and, combined with the sensitization effect of BP, achieved ultra-high-sensitivity detection of the Au@cDNA. The optical biosensor constructed in this study has the potential for detecting low-concentration target sequences and holds great development prospects in the field of biosensing research.

## Figures and Tables

**Table 1:** BRCA1 synthetic sequence.

BRCA1 Biomolecules	Sequence (5′–3′)
Probe DNA (pDNA)	AAGTATCAGGGTAGTTCTGTCAAACTTGCATGTGGAGCCA
Complementary DNA (cDNA)	TGGCTCCACATGCAAGTTTGACAGAACTACCCTGATACTT-(CH_2_)_6_-SH
Non-Complementary DNA (N-cDNA)	TTTATCGGTAATCCGGTTATTGCCATGGGGATTCCAGGTT-(CH_2_)_6_-SH

**Table 2:** Performance comparison of BRCA1 detection based on different types of sensors.

Sensor Type	Detection Principle	Sample Type	Detection Time	LOD	Ref.
Electrochemical detection based on signal amplification of gold nanoparticles	Fluorescence PCR (KASP)	Clinical whole blood	<3 h	1 fM	[27]
Multi-walled CNT (MWCNT)-modified glassy carbon electrode (GCE)	Potentiometric stripping analysis	Synthetic oligonucleotide	20 min	100 fM	[28]
Single-walled carbon nanotube (SWCNT)-based screen-printed graphite electrodes	Differential pulse voltammetry (DPV)	Synthetic oligonucleotide	30 min	378.52 nM	[29]
Genomagnetic electrochemical assay	Potentiometric stripping analysis	Synthetic oligonucleotide	~30 min	160 pM	[30]
STMS sensor based on signal amplification of gold nanoparticles	Mach-Zehnder interferometry	Synthetic oligonucleotide	60 min	20.27 fM	This Work

## Data Availability

All data supporting the findings of this study are included within the manuscript.
